# Differential diagnosis of syndromic craniosynostosis: a case series

**DOI:** 10.1007/s00404-021-06263-9

**Published:** 2021-10-11

**Authors:** Tamara Casteleyn, Denise Horn, Wolfgang Henrich, Stefan Verlohren

**Affiliations:** 1 Department of Gynecology and Obstetrics, Sana Klinikum Lichtenberg, Berlin, Germany; 2 Institute of Medical Genetics and Human Genetics, Charité – Universitätsmedizin, Berlin, Germany; 3 Department of Obstetrics, Charité – Universitätsmedizin, Berlin, Germany

**Keywords:** Syndromic craniosynostosis, Apert syndrome, Saethre Chotzen syndrome, Prenatal ultrasound

## Abstract

**Purpose:**

Syndromic craniosynostosis is a rare genetic disease caused by premature fusion of one or multiple cranial sutures combined with malformations of other organs. The aim of this publication is to investigate sonographic signs of different syndromic craniosynostoses and associated malformations to facilitate a precise and early diagnosis.

**Methods:**

We identified in the period of 2000–2019 thirteen cases with a prenatal suspected diagnosis of syndromic craniosynostosis at our department. We analyzed the ultrasound findings, MRI scans, genetic results as well as the mode of delivery, and postnatal procedures.

**Results:**

Eight children were diagnosed with Apert Syndrome, two with Saethre Chotzen syndrome, one with Crouzon syndrome, and one with Greig cephalopolysyndactyly syndrome. One child had a mutation p.(Pro253Leu) in the FGFR2 gene. We identified characteristic changes of the head shape as well as typical associated malformations.

**Conclusion:**

Second trimester diagnosis of syndromic craniosynostosis is feasible based on the identified sonographic signs. In case of a suspected diagnosis a genetic, neonatal as well as surgical counseling is recommended. We also recommend to offer a fetal MRI. The delivery should be planned in a perinatal center.

## Introduction

Craniosynostosis is the result of a premature fusion of one or multiple cranial sutures. Depending on the affected sutures, the head can develop asymmetrically which is detectable in in utero with prenatal ultrasound. Postnatally, surgery may be necessary in case of an increase in intracranial pressure.

Isolated craniosynostosis is mostly sporadic, with an incidence of 1:2000–2500 [1]. In contrast, syndromic craniosynostosis usually involves multiple sutures combined with malformations of other organs [2]. Syndromes most frequently associated with craniosynostosis are Apert-, Crouzon-, Pfeiffer-, and Saethre Chotzen syndrome. Of these, the Apert syndrome is the most common with a prevalence of 1:100,000 [3]. A mutation of Fibroblast Growth Factor Receptor 2 (FGFR2) gene causes the autosomal dominantly inherited Apert syndrome [4]. However, most individuals with Apert syndrome develop the disorder as the result of a de novo mutation in the FGFR2 gene.

The Apert syndrome causes variable deformation of the skull due to bicoronal craniosynostosis, midface hypoplasia and complex syndactyly of hands and feet [5, 6]. It can also be associated with the central nervous system's abnormalities, including malformations of the corpus callosum, the limbic system and abnormal gyration [7]. Neurological development disorders are possible that mostly lead to mild and rarely moderate to severe impairment [6]. After birth, high intracranial pressure can indicate a need for surgery.

The autosomal dominant Crouzon syndrome is also caused by mutations in FGFR2 gene [8]. Similar to Apert syndrome, the clinical features of Crouzon syndrome are a tall, flattened forehead caused by bicoronal craniosynostosis, and midface hypoplasia. The degree of the malformations is milder, and limbs are usually not affected [9]. Bicoronal craniosynostosis and syndactyly characterize Pfeiffer syndrome that is caused by autosomal dominant mutations of FGFR1- or FGFR2 gene [10, 11]. Additional sutures can be affected, and skeletal, central nervous system and gastrointestinal abnormalities can occur [12]. Saethre Chotzen syndrome is characterized by mild craniosynostosis of different cranial sutures and syndactyly and is caused by the autosomal dominant mutation of TWIST gene and the FGFR2 gene [13, 14].

The focus of our case series is to identify the contribution of prenatal ultrasound for an early and precise prenatal diagnosis of syndromic craniosynostosis [5]. In case of a suspected diagnosis, genetic counselling and testing including the newest methods of whole genome/exome sequencing and/or targeted panel diagnosis is recommended. A precise differentiation between syndromic and nonsyndromic causes is paramount to allow for specific counselling [15]. A fetal MRI should also be performed to confirm the diagnosis and identify possible central nervous malformations [5].

We aim to describe the prenatal sonographic signs and their contribution in the diagnostic work up in cases with suspected syndromic craniosynostoses. We aim to raise awareness for this disease complex to facilitate a precise and early diagnosis which is essential for perinatal management and the interdisciplinary counseling of the parents.

## Materials and methods

We identified thirteen cases of syndromic craniosynostosis in the Viewpoint (GE, Solingen, Germany) and the SAP (Walldorf, Germany) patient databases of the Department of Obstetrics and the Department of Pediatric Surgery at Charité—Universitätsmedizin Berlin in the years 2000–2019. We searched for keywords indicating abnormal biometric parameters of the head, brain anomalies or both, and eventful findings of the limbs. We furthermore used exact keyword search for the keywords Apert syndrome, craniosynostosis, Crouzon-, Saethre Chotzen- or Pfeiffer syndrome.

This search resulted in 389 cases of abnormal findings. A subsequent manual review identified syndromic craniosynostosis in thirteen fetuses.

In addition, we compared the results with the surgery records of the Department of Pediatric Neurosurgery. No additional cases were identified. After retrieving the patients, we analyzed ultrasound findings, MRI scans, genetic results and the mode of delivery and postnatal procedures.

## Results

Between 2000 and 2019, we identified thirteen pregnancies with high suspicion of syndromic craniosynostosis in our department. A detailed description of the sonographic findings is found in Table 1. In ten cases, Apert syndrome was suspected due to specific sonographic features. Molecular genetic testing revealed a p.(Pro253Arg) mutation in the FGFR2 gene and confirmed the diagnosis in five cases. However, in one case, the postnatal genetic test detected a mutation in *GLI3*-gene, which causes Greig cephalopolysyndactyly syndrome, which is associated with craniosynostosis [16]. In another case, the genetic test revealed a p.(Pro253Leu) mutation in the FGFR2 gene. The subsequent tests of the parents identified the same mutation in the father who had not been diagnosed previously. Three patients did not give consent for genetic testing.

**Table 1:** Sonographic findings in syndromic craniosynostosis

Syndrome	Sonographic findings
Apert syndrome (*n* = 8)	Syndactyly (8/8) Frontal bossing (5/8) Cloverleaf skull (4/8) Turricephaly (2/8) Dolichocephaly (1/8) Polyhydramnios (1/8) A. lusoria dextra (1/8) Mild ventriculomegaly (1/8) Dysgenesis of corpus callosum (1/8) Cleft palate (1/8) Retracted bridge of the nose (1/8)
Saethre Chotzen syndrome (*n* = 2)	Turricephaly (1/2) Saddle nose (1/2) Flat facial profile (1/2)
Crouzon syndrome (*n* = 1)	Flattened occiput Depressed frontoparietal bones Protruded bulbi Small thorax with short ribs
Greig cephalopolysyndactyly syndrome (*n* = 1)	Agenesis of corpus callosum Hypertelorism Right-sided aortic arch Polydactyly

Two children were diagnosed with Saethre Chotzen syndrome, one child with Crouzon syndrome. The gestational age when the diagnosis was suspected was between 20 + 1 and 33 + 4 weeks of gestation. Nine patients received the diagnosis in the second trimester, four patients in the third trimester. In all cases after 2017, we recommended a fetal MRI. This was conducted in three cases and confirmed the sonographic results.

**Fig. 1:**
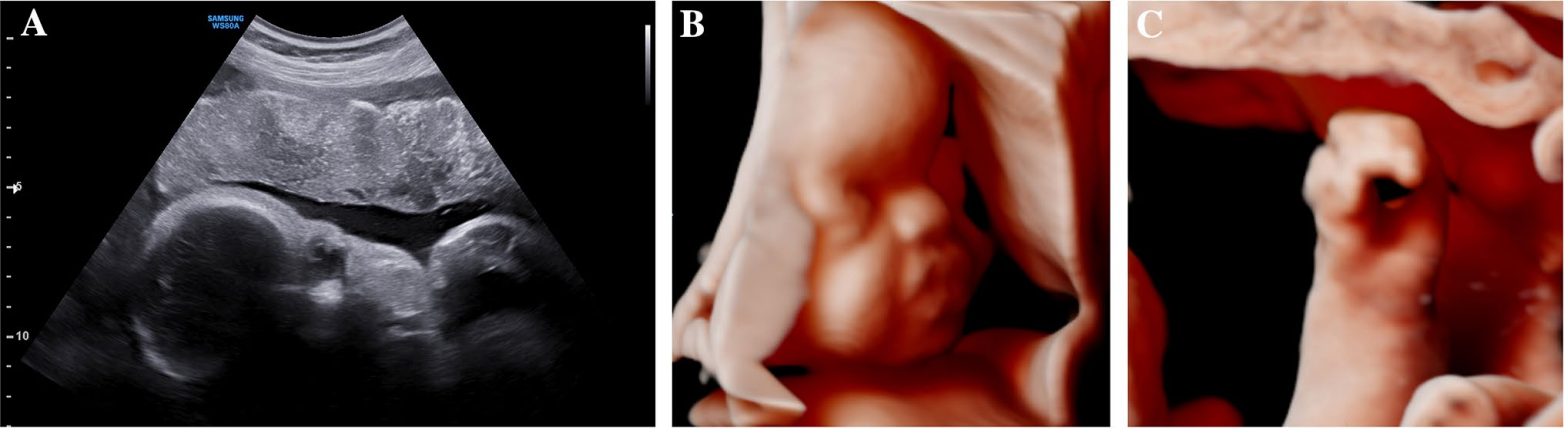
Fetus with Apert syndrome in 30 + 3 weeks of gestation. The ultrasound examination shows a prominent forehead with frontal bossing (**a**, **b**). Bilateral syndactyly can be imaged (**c**)

**Fig. 2:**
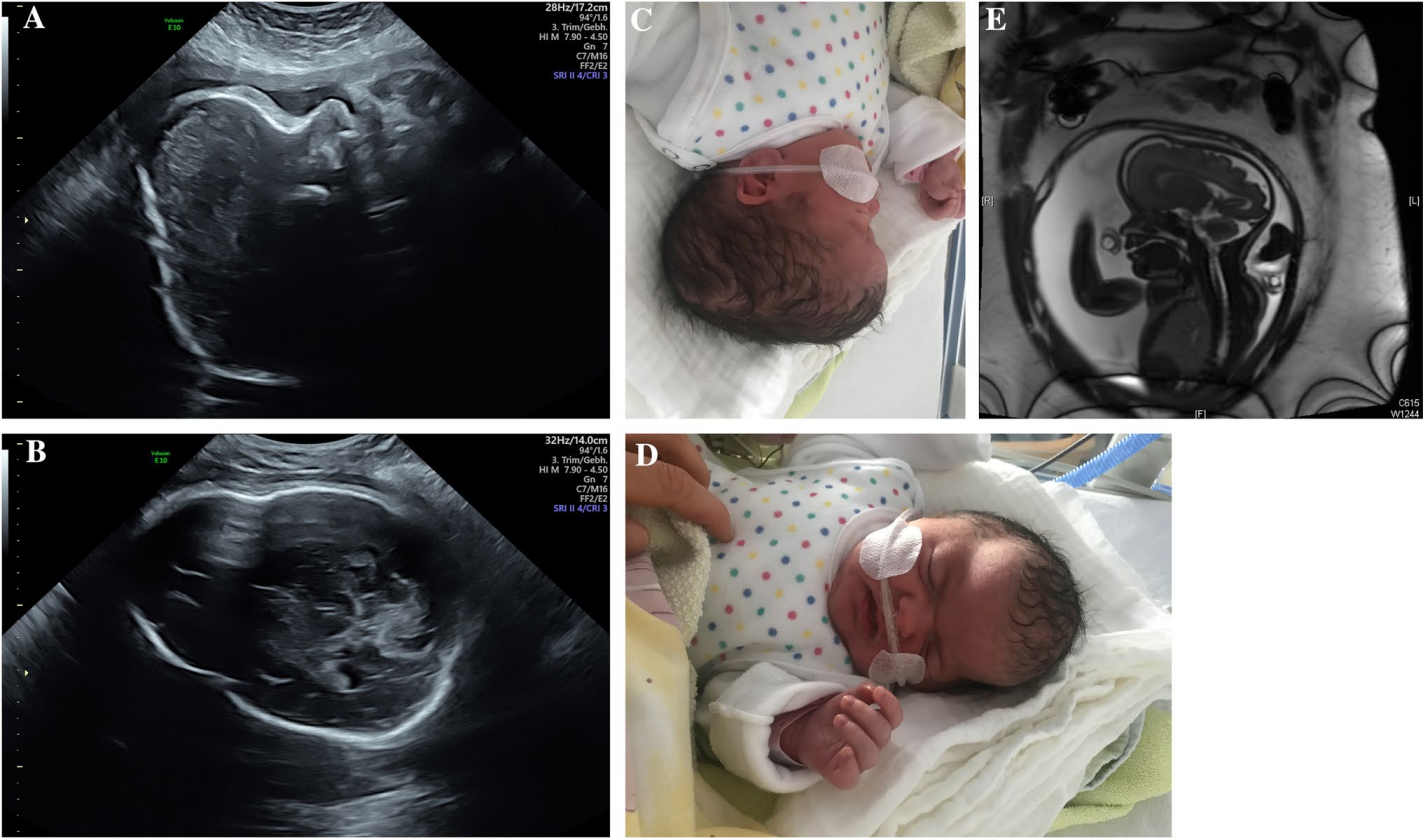
Child with suspected Apert syndrome. The prenatal ultrasound exam in 31 + 3 weeks of gestation shows a prominent shape of the skull with bicoronal and sagittal craniosynostosis as well as frontal bossing (**a**, **c**). The prenatal MRI confirms the findings (**e**). After the birth, a scaphocephaly with a long and narrow skull and high forehead is seen (**d**). The genetic examination showed a mutation in FGFR2-gene (Pro253Leu), the father had the same mutation. At this amino acid position is the pathogen mutation p.Pro253Arg located, which leads to Apert syndrome

In the fetuses with Apert syndrome, typical sonographic features were frontal bossing (5/8 cases) as well as a cloverleaf skull (4/8) (Table 1 and Figs. 1a, b, 2a–e). In two cases, the examiner described a turricephaly, a tall head shape caused by coronal craniosynostosis (Tables 1, 2). In all cases, a prenatal ultrasound revealed a diagnosis of syndactyly (Fig. 1c).

**Table 2:** Overview of malformations in syndromic craniosynostosis

Syndrome	Affected sutures	Head	Associated malformations
Apert syndrome	Usually multiple sutures, especially bilateral coronal sutures and variable other sutures [5, 6]	Acrobrachycephaly, flat occiput, hypertelorism, flat forehead, midface hypoplasia, asymmetric cranial shape [24], turricephaly [5]	Syndactyly, central nervous malformations (dysgenesis of corpus callosum, abnormal gyration) [5, 24], neurological development disorders possible
Crouzon syndrome	Variable; often coronal and sagittal sutures [24]	Acrobrachycephaly, (symmetric) midface hypoplasia, hypertelorism [24],	Extremities not involved, no neurological development disorders
Saethre Chotzen syndrome	Uni-/bilateral coronal sutures, other sutures possible [25]	Plagiocephaly, brachycephaly, acrocephaly, deep hairline, small ears, high forehead, asymmetric face (overall milder than Apert syndrome) [25]	Syndactyly possible, brachydactyly, rarely neurological development disorders, skeletal and cardiac malformations [25]
Greig cephalopoly- syndactyly syndrome	Frontal und sagittal sutures [16]	Macrocephaly, prominent forehead, hypertelorism [26], trigonocephaly [16]	Polydactyly, broad thumbs/big toes, cutaneous syndactyly, neurological development disorders possible [26], dysgenesis of corpus callosum [16]
Pfeiffer syndrome	Bilateral coronal- und lambdoidal sutures, rarely sagittal suture [27]	Flat occiput, high forehead, hypertelorism, cloverleaf skull, variable midface hypoplasia [12, 27]	Broad thumbs/big toes with deviation, syndactyly possible, neurological development disorders possible [12, 27]

In two separate Saethre Chotzen syndrome cases, the diagnosis of a bicoronal craniosynostosis occurred in one case before birth, along with identification of a saddle nose and a flat profile (Table 1). The diagnosis of syndactyly of hands and feet occurred after birth. In the second fetus with Saethre Chotzen syndrome, the only anomaly detected was a prominent forehead.

**Fig. 3:**
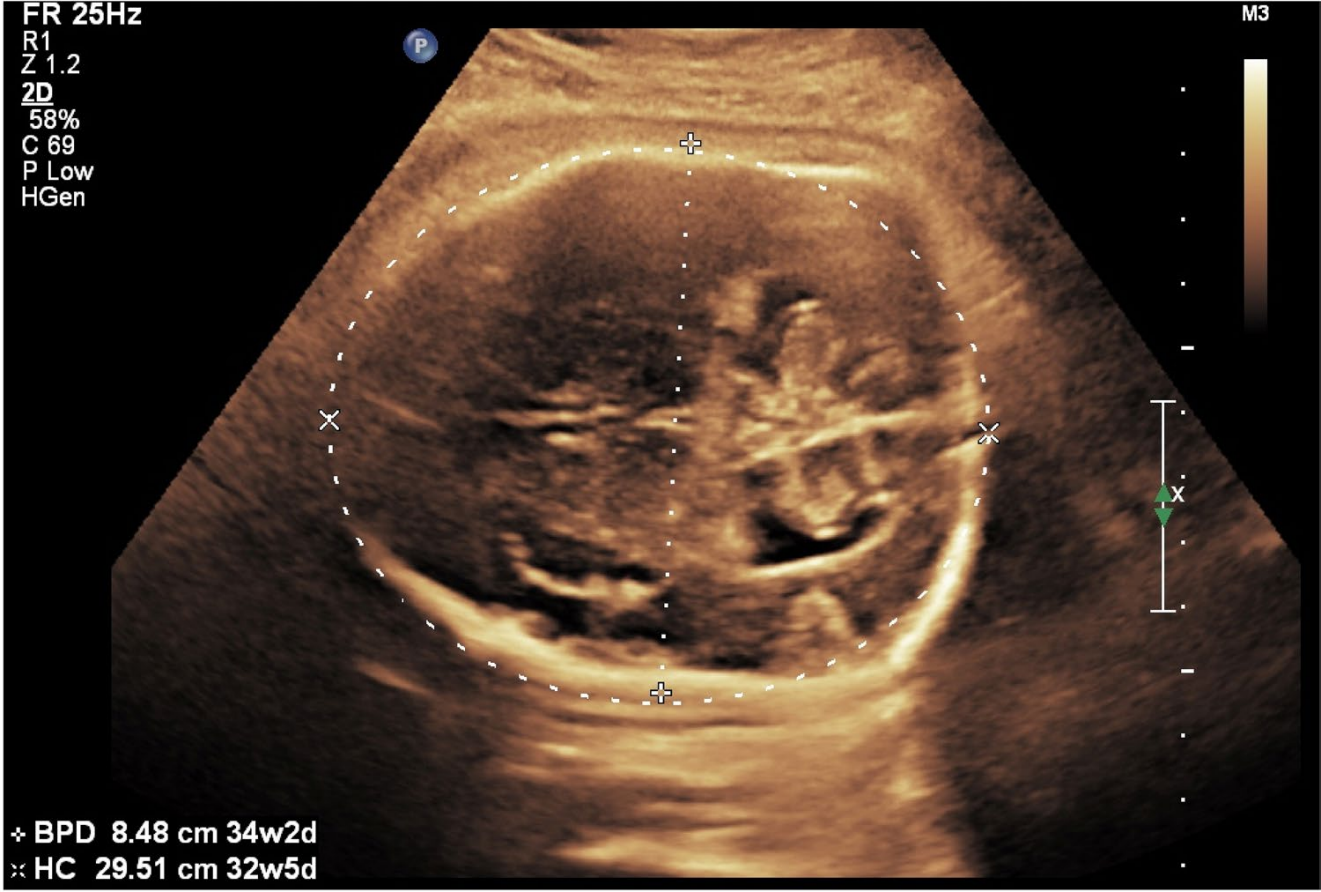
Fetus with bilateral coronal synostosis caused by Crouzon syndrome. The ultrasound shows a flattened occiput and mild bilateral frontal depressions of the skull

The patient with fetal Crouzon syndrome presented with an abnormal shape of the head with a flat occiput, depressed frontoparietal bones (Fig. 3) and protruding eyes, and a small thorax with short ribs (Table 1).

**Fig. 4:**
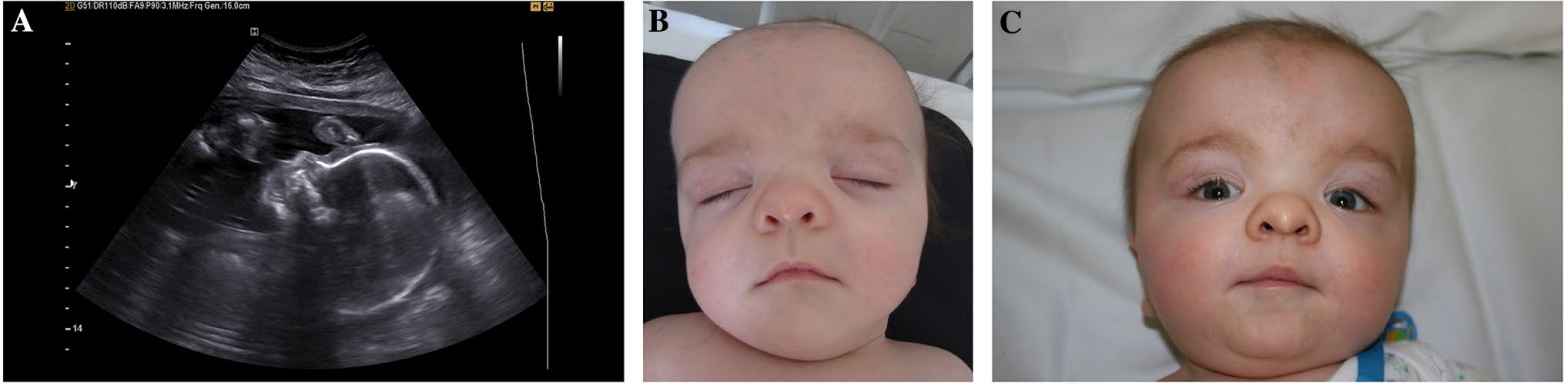
Sonographic and postnatal images of a child with Greig cephalopolysyndactyly syndrome. The cranial biometric parameters were in the normal range, a dysgenesis of the corpus callosum was suspected. After the birth, a high forehead with down-slanting palpebral fissures and a low nose root is seen

**Fig. 5:**
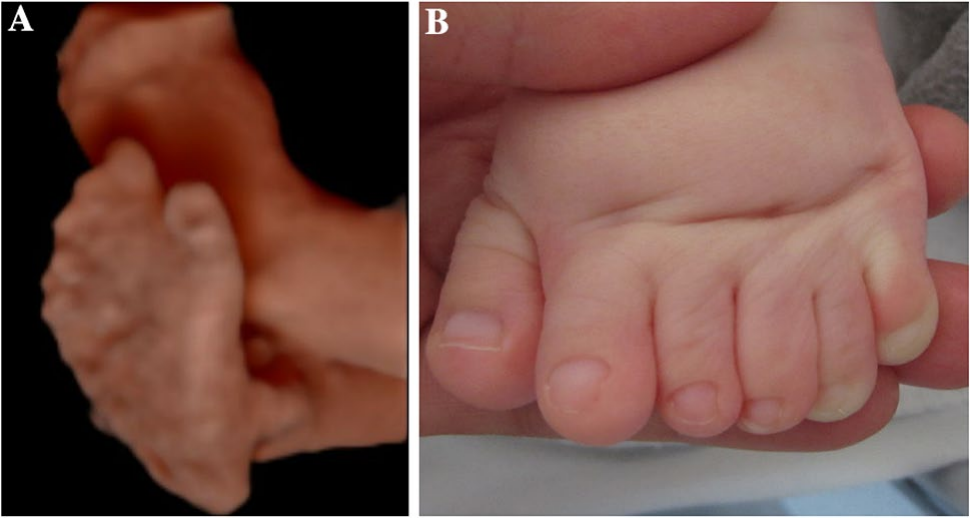
Child with Greig cephalopolysyndactyly syndrome. The sonographic diagnosis of postaxial polysyndactyly (**a**) was confirmed after the birth (**b**)

The fetus with Greig cephalopolysyndactyly syndrome exhibited hypertelorism, agenesis of the corpus callosum, a right-sided aortic arch, and polydactyly (Table 1, Figs. 4a, 5). Postnatally, these findings were confirmed (Figs. 4b, c, 5b); furthermore, additional identification of malformations in the child with Greig cephalopolysyndactyly syndrome included a subaortic ventricular septal defect and a deformation of the feet.

After the prenatal diagnosis of a fetal syndromic craniosynostosis and after extensive interdisciplinary counselling, seven couples decided to terminate the pregnancy. In all of these cases, a fetal Apert syndrome was diagnosed. Of these couples, four decided for an autopsy of the fetus. The pathoanatomical examinations and the radiological fetograms confirmed the sonographic findings. Of the other children, five were delivered by cesarean section and one child was delivered through vaginal birth. In one case the cesarean section was indicated due to fetal breech position. Three patients decided to have a preventive cesarean section and one patient had a repeat cesarean section.

For analyzing the long-term postnatal outcome, the data of six children who were treated at our department for pediatric neurosurgery was available. One child (born 2011) with Crouzon syndrome received a decompressive craniectomy in his first year of life due to high intracranial pressure. Following that multiple surgeries (last in 2020) for fronto-orbital remodeling and multiple corrections of craniofacial defects were performed. This child also suffers a hearing loss due to aural atresia and subsequent speech development disorder.

Of the two children diagnosed with Saethre Chotzen syndrome one (born 2014) underwent fronto-orbital remodeling ten months after birth. The other one (born 2019) was treated with a strip craniectomy four months after birth. As of now, no surgery was indicated for the child with Apert syndrome (born 2019). The child diagnosed with the p.(Pro253Leu) mutation (born 2019, Fig. 1) had a biparietal strip craniectomy two months after birth due to raised intracranial pressure. The child with Greig cephalopolysyndactyly syndrome had a foot surgery to correct the hexadactyly and the pes supinatus (Fig. 5).

## Discussion

**Fig. 6:**
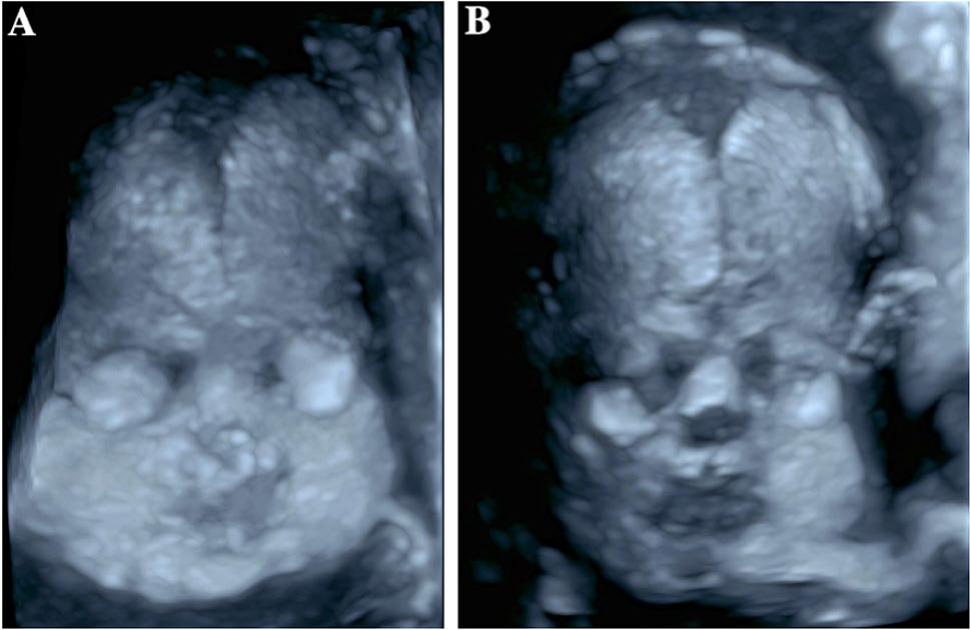
Fetus with normal face and skull shape imaged by three-dimensional skeletal imaging mode in 27 + 5 weeks of gestation

**Fig. 7:**
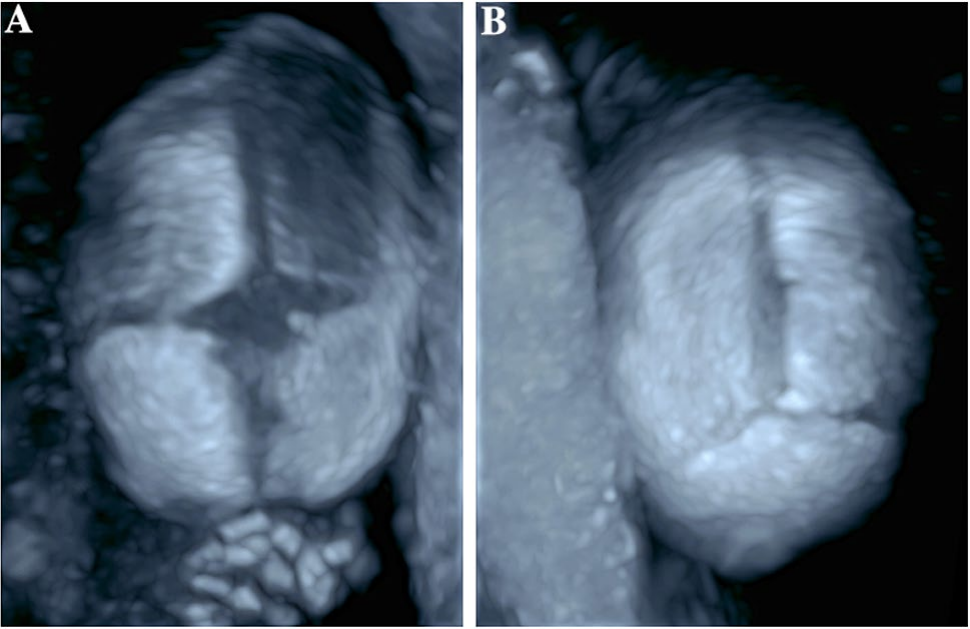
Normal findings of a fetal skull imaged by three-dimensional ultrasonic skeletal imaging mode. Image of the anterior fontanelle with adjacent sutures—sutura frontalis, sutura sagittalis and sutura coronalis (**a**). Image of the posterior fontanelle with adjacent sutures—sutura sagittalis, sutura lambdoidea (**b**)

**Fig. 8:**
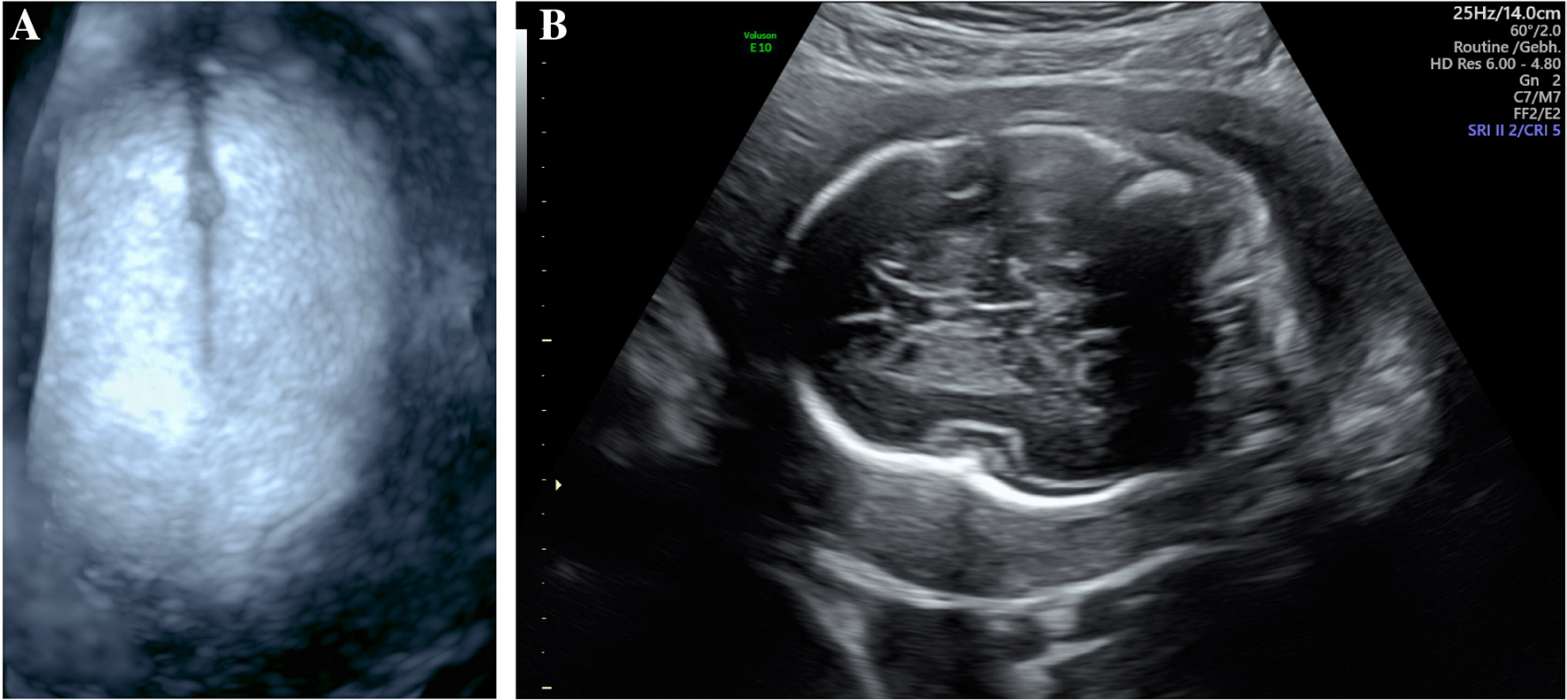
Fetus with partial sagittal craniosynostosis in 23 + 4 weeks of gestation. By three-dimensional ultrasonic skeletal imaging mode, a partial premature fusion of the sagittal suture can be shown (**a**). The B-mode image shows a prominent shape of the skull, a sagittal craniosynostosis is suspected (**b**)

Syndromic craniosynostosis is a rare disease complex that shows characteristic features detectable in prenatal ultrasound. An early precise diagnosis is important for the interdisciplinary counseling of the parents and the perinatal management. Our study confirms that a prenatal detection of syndromic craniosynostosis in the second trimester is possible. In our case series, the diagnosis was suspected at the time of the second trimester screening in 9/13 patients and in 4/13 patients between 27 + 0 and 33 + 4 weeks of gestation. However, the diagnosis can be challenging as the extent of the skull deformity can vary and the standardized measurements of the head (biparietal diameter and head circumference) are not necessarily outside the normal range [17]. To confirm the diagnosis, it can be helpful to use a three-dimensional ultrasonic skeletal imaging mode to image the skull with the sutures (Figs. 6, 7, 8). Though diagnosis is mostly feasible without using the skeleton mode, as we have used B-mode and conventional 3D ultrasound in our cases to establish the diagnosis.

### Value of sonographic signs: skull shapes

In accordance to the literature, an abnormal shape of the skull was the leading sonographic sign in our case series. The basic biometric parameters may be out of range. Depending on the affected sutures the biparietal diameter (BPD) and the cephalic index (CI) can be raised or lower [17].

In our cohort, all cases with Apert syndrome exhibited an abnormal shape of the skull (Figs. 1, 2). We detected frontal bossing in 5/8 cases, a cloverleaf skull in 4/8 cases, a turricephaly in 2/8 cases and a dolichocephaly in 1/8 cases (see Table 1). In one of the fetuses with Apert syndrome, agenesis of corpus callosum was diagnosed. Malformations of midline structures like dysgenesis of the corpus callosum are reported in up to 11% as well as alterations of the temporal lobe [7, 18].

An abnormal shape of the skull was the leading ultrasonographic finding also in the other cases of syndromic craniosynostosis. In one case of Saethre Chotzen syndrome, the fetus presented with turricephaly with flat profile and saddle nose. In the other case the head was small without other sonomorphological alterations. In the fetus with Greig cephalopolysyndactyly syndrome a hypertelorism and agenesis of corpus callosum were noted and the fetus with Crouzon syndrome had protruding bulbi of the eyes.

In accordance to other published case series the diagnosis of syndromic craniosynostosis was suspected in most cases in the second trimester as the skull deformation develops at this time [14]. Owing to premature fusion of sutures, the underlying structures are harder to visualize. This effect has been identified as an early indirect sign for craniosynostosis called `brain shadowing sign’. It can be noted prior to changes of the skull shape and is also reported in fetuses with mild changes [19]. In this retrospective study, cases of syndromic craniosynostosis were detected in 25 weeks of gestation. However, the brain shadowing sign is not specific for craniosynostosis and can also be seen in fetal head molding or open spina bifida. The authors also report that in accordance to our results an abnormal head shape was seen in 23 of 24 cases. Other signs were facial abnormalities, syndactyly and ventriculomegaly [19]. Furthermore, the cranial bones with the sutures and facial alterations can be seen more detailed with three-dimensional ultrasound, especially with the feature “skeleton mode”, as compared to B-mode (Figs. 6, 7, 8) [20]. In the skeleton mode, the premature fusion of the suture can be imaged exactly (Fig. 8).

### Value of sonographic signs: other malformations

Other organ malformations may occur in syndromic craniosynostosis. A thorough sonographic examination of hands and feet is mandatory, as malformations of extremities are common and may be used to distinguish between isolated and syndromic craniosynostosis. The type of limb abnormalities indicates which type of syndromic craniosynostosis is likely. In accordance to the literature, in all our cases of Apert syndrome, syndactyly of upper and partly of the lower extremities were diagnosed by ultrasound (Table 1 and Fig. 1c) [17, 19, 20]. In one case of fetal Saethre Chotzen syndrome, syndactyly was diagnosed only postnatally. In another case series, anal atresia and a patent ductus arteriosus were detected postnatally in a child with Saethre Chotzen syndrome [19]. In accordance to Hurst et al. we diagnosed a polydactyly in a fetus with Greig cephalopolysyndactyly syndrome [16]. Besides the thorough sonographic assessment of the skull, the central nervous system and limbs, it is important to examine the other organ systems as well to detect possible accompanied malformations which might have an impact on the child’s prognosis. Our case of Greig cephalopolysyndactyly syndrome was associated with a right aortic arch. A subaortic ventricular septal defect was additionally diagnosed postnatally. In the literature, congenital heart defects are described in Greig cephalopolysyndactyly syndrome and include ventricular septal defects, atrial septal defects and patent ductus arteriosus as well as double outlet right ventricle [16]. Congenital heart defects in combination with craniosynostosis and polydactyly can also be seen in Pfeiffer syndrome and the rare Carpenter syndrome [21]. The rare Antley Bixler syndrome is characterized by craniosynostosis, humero-radial synostosis, a curved femur and contractures of the joints. Cardiac and urogenital defects are possible [22].

### Value of fetal MRI

In our case series, a fetal MRI was performed in three cases. This examination confirmed the sonographic findings and identified no further abnormalities of the central nervous system (Fig. 2e). However, a study of Rubio et al., compared the results of ultrasound exams and fetal MRI after the diagnosis of syndromic craniosynostosis. The MRI detected two cases of dysgenesis of corpus callosum and one tethered cord syndrome that were not detected with ultrasound [5]. Malformations of the central nervous system can cause neurodevelopmental disorders and are important when counseling the parents. In conclusion, a fetal MRI should be offered to all patients with suspected syndromic craniosynostosis. However, a precise prenatal prognosis regarding developmental disorders is not possible.

### Value of molecular genetic tests

Genetic testing must be offered to the patients in order to distinguish between isolated and syndromic craniosynostosis and to confirm the entity of the craniosynostosis. A genetic examination of the parents can be performed since not only sporadic mutations, but also autosomal dominant inheritance with variable symptoms is possible. In our study, one parent was previously diagnosed with Saethre Chotzen syndrome. In another case of suspected Apert syndrome a genetic testing was conducted. The result showed a p.(Pro253Leu) mutation in the FGFR2 gene not only in the DNA of the fetus, but also in the father’s DNA who had not been diagnosed with craniosynostosis previously.

### Mode of delivery

In our case series, five patients had a cesarean section and one patient a vaginal birth. Similar to our results Harada et al., described a high rate of cesarean sections (73%) in patients with fetal craniosynostosis [17]. If the fetus is in cephalic position and the head circumference is not raised excessively there is no absolute indication for a cesarean section. The patients should be informed about the higher risk of arrested labor and emergency cesarean section in case of significant skull deformities. The delivery should be planned in a perinatal center to assure an ideal postnatal care of the newborn especially regarding the airway management. Prenatally a Pediatric Neurosurgeon should be consulted and inform the parents about possible operative procedures.

After birth a cranial ultrasound and a cranial MRI should be performed. Owing to the higher prevalence of cardiac defects an echocardiography is recommended as well as an examination by a pediatric surgeon.

### Limitations

Due to the low incidences of syndromic craniosynostosis we could only analyze thirteen cases with suspected craniosynostosis. Another limitation is the retrospective character of the study. A comparison between ultrasound and MRI is limited as we did not perform an MRI in all cases.

## Conclusion

In conclusion, syndromic craniosynostosis is a rare disease. A prenatal diagnosis in the second trimester is feasible based on the sonographic signs described here. An early and precise diagnosis should be achieved to allow for targeted counselling. In case of a suspected diagnosis a genetic, neonatal and surgical workup is recommended and a fetal MRI should be conducted. Owing to midface hypoplasia and alterations of the upper airway the newborns can be respiratory compromised. Consequently the delivery should be planned in a perinatal center [23].

## Data Availability

The data available on request from the corresponding author.
